# Potential biological role of poly (ADP-ribose) polymerase (PARP) in male gametes

**DOI:** 10.1186/1477-7827-7-143

**Published:** 2009-12-05

**Authors:** Ashok Agarwal, Reda Z Mahfouz, Rakesh K Sharma, Oli Sarkar, Devna Mangrola, Premendu P Mathur

**Affiliations:** 1 Center for Reproductive Medicine, Cleveland Clinic, Cleveland, OH, USA; 2 Department of Biochemistry and Molecular Biology, Pondicherry University, Pondicherry, India; 3 McGill University Health Center, Montreal, Canada

## Abstract

Maintaining the integrity of sperm DNA is vital to reproduction and male fertility. Sperm contain a number of molecules and pathways for the repair of base excision, base mismatches and DNA strand breaks. The presence of Poly (ADP-ribose) polymerase (PARP), a DNA repair enzyme, and its homologues has recently been shown in male germ cells, specifically during stage VII of spermatogenesis. High PARP expression has been reported in mature spermatozoa and in proven fertile men. Whenever there are strand breaks in sperm DNA due to oxidative stress, chromatin remodeling or cell death, PARP is activated. However, the cleavage of PARP by caspase-3 inactivates it and inhibits PARP's DNA-repairing abilities. Therefore, cleaved PARP (cPARP) may be considered a marker of apoptosis. The presence of higher levels of cPARP in sperm of infertile men adds a new proof for the correlation between apoptosis and male infertility. This review describes the possible biological significance of PARP in mammalian cells with the focus on male reproduction. The review elaborates on the role played by PARP during spermatogenesis, sperm maturation in ejaculated spermatozoa and the potential role of PARP as new marker of sperm damage. PARP could provide new strategies to preserve fertility in cancer patients subjected to genotoxic stresses and may be a key to better male reproductive health.

## Background

Male fertility is affected by a variety of environmental, behavioral, and genetic factors that can alter spermatogenesis at various levels [1-3]. Male germ cells are exposed to a wide variety of endogenous and exogenous genotoxic agents. Endogenous agents include reactive oxygen and nitrogen species generated during the metabolic activities of cells [4,5]. Exogenous agents include various environmental factors that can inflict damage to genomic DNA. These genotoxic agents can introduce DNA lesions in the form of DNA single and double strand breaks, abasic sites, base damage, inter-and intra-strand cross links and DNA-protein cross links [6,7]. Origin of DNA damage in human spermatozoa can occur by abortive apoptosis, abnormal chromatin packaging, generation of reactive oxygen species and premature release from Sertoli cells [8-12].

During spermatogenesis, germ cell DNA in the nucleus is nicked by topoisomerases in order to relieve the torsional stress created when DNA is compacted into the differentiating sperm head. Persistence of DNA strand breaks during different stages of spermatogenesis contribute to DNA damage detected in mature spermatozoa [13,14]. As spermatids are haploid, they must resolve double stranded DNA breaks by an error-prone DNA repair mechanism [15]. Interest in male germ cell DNA quality has increased in recent decades especially in the era of assisted reproductive technologies (ART). As natural selection of spermatozoa for fertilization is bypassed in procedures such as intracytoplasmic sperm injection (ICSI), awareness has been raised regarding the possibility of congenital anomalies. Many reviews have dealt with the origin of sperm DNA integrity, evaluation of available technologies to assess sperm DNA integrity and its impact on the outcome of ART [16-24]. Environmental, life style and occupational hazards in male infertility have also been extensively studied [25-33]. These factors may affect DNA repair pathways and impact male fertility and subsequent embryo development.

In this review we will discuss the role of Poly (ADP-ribose) polymerase (PARP), a DNA damage repair proteins and highlight its role in ejaculated human spermatozoa. Recently the focus of PARP's role in malignancy has intensified to include use of PARP inhibition as an adjuvant therapy with chemotherapeutic drugs [34]. As our interest lies primarily in male reproductive health, in this review we will focus on the biological role of PARP in general as well as in male gamete and highlight possible role of PARP in modulating DNA damage in male germ cells.

### Poly (ADP-ribose) polymerase (PARP)

Poly (ADP-ribose) polymerase (PARP), a nuclear enzyme has a particularly well-researched role in base excision repair; it is one of the primary repair mechanisms to resolve DNA lesions caused by endogenous processes as well as those caused by exogenous chemical exposure and irradiation [35,36]. PARP also has a well-documented role in testicular germ cells [37-39], including a role in DNA damage repair of germ cells [40]. However, a similar role for PARP in human ejaculated spermatozoa is still being investigated. The last decade has seen increasing interest in the relationship between DNA integrity in mature ejaculated spermatozoa and male infertility [16,26,41-43]. Focus on genomic integrity of the male gametes has been further intensified by the growing concern about the transmission of genetic diseases through intracytoplasmic sperm injection (ICSI) [44-49].

Proteins involved in the major repair pathways have been shown to be expressed in the testis [50]. PARP proteins are involved in detection of strand breaks and signaling in both the base excision repair and nucleotide repair pathways [51,52]. PARP catalyzes poly (ADP-ribose) (PAR) polymerization from donor NAD^+ ^molecules into target proteins. PARP1 is the prototype and most abundantly expressed member of a family of PARPs.

The PARP family consists of 18 homologues (PARP 1-18) with a conserved catalytic domain made up of 50 amino acid residues that serve as the 'PARP signature' [53]. This is the site where poly(ADP-ribose) (PAR) chains are initiated, elongated, and where branching of the chains can occur [54]. Besides this catalytic domain, PARP family members may also have other domains including DNA binding domains, macro-domains, breast cancer-1 (BRCA-1) C-Terminus (C-T) domain, ankyrin repeats and a domain associated with protein-protein interaction called WWE. BRCA-1 C-T domains are characteristic of proteins responding to DNA damage at cell cycle checkpoints while WWE domains are found in proteins associated with ubiquitination. All of these special types of domains contribute to the unique functions of each family member [53,55,56].

PARP family members can be divided into several subcategories or groups based on each protein's established functional domains and precise functions into: 1) DNA dependent PARPs (PARP1 and PARP2) that are activated by DNA strand breaks 2) Tankyrases (tankyrase-1 and tankyrase-2) that serve diverse functions such as telomere regulation and mitotic segregation 3) CCCH-type PARPs (PARP12, PARP13) which contain special CCCH type zinc fingers and 4) PARP9, PARP14, and PARP15 consisting of macro PARP's which have 1-3 macrodomains connected to a PARP domain. They also have WWE domain and PARP catalytic activity. PARP6, 8, 11 and 16 do not have any recognized domains or functions and therefore they have not been assigned proper nomenclature [56].

Recent classification system by Hassa and Hottiger groups PARPs on the basis of their catalytic domain sequences [54]. PARP family is divided into 3 separate groups: 1) PARP1, PARPb (short PARP1), PARP2, and PARP3, 2) PARP4 and 3) 2 PARP members, Tankyrase-1, tankyrase-2a, and its isoform tankyrase-2b (also known as PARP5 and PARP6a/b) [54]. The various PARP enzymes can also have different subcellular localization patterns. PARP1 and 2 are considered nuclear enzymes and are found in the nucleus of cells. In contrast, tankyrases and PARP3 are found in both the nucleus and cytoplasm [54,57].

**Figure 1:**
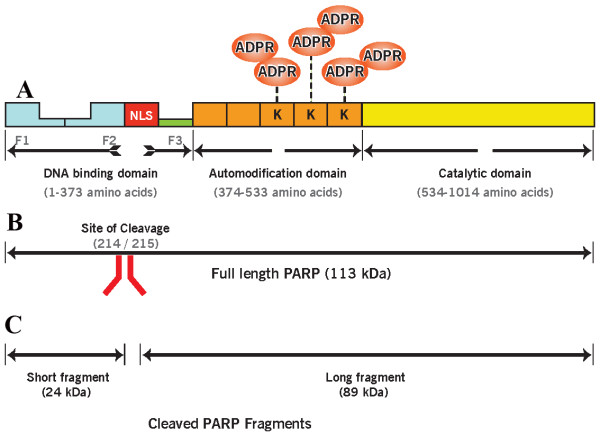
**Structural domains of PARP and its fragments showing**. A: DNA binding domain containing Zinc fingers (F1-F3) for nucleosome binding and nuclear localization (NLS) segment; Automodification domain responsible for adding ADPR (ADP ribose) polymers through binding with Lysine (K) amino acid and catalytic domain has the PARP signature and PARP enzymatic activity. B: Full length PARP1 113 KDa molecule with a mark on the site of cleavage (214/215 amino acids) C: PARP cleavage by caspase showing short (24 KDa) and long (89 KDa) cleaved PARP fragments.

Perhaps the best studied member of the PARP family is PARP1, a 113 kD enzyme encoded by the ADP-ribosyl transferase (ADPRT) gene in humans located on chromosome 1 [58,59]. PARP1 has been reported to be involved in regulation of chromatin structure and transcription processes in response to specific signaling pathways [55]. The protein structure of PARP1 is well characterized. Figure 1 represents the PARP1 structure domains with clarification on the sites of the zinc fingers, PAR acceptance, and cleavage site with short and long cleaved PARP1 fragments. PARP1 is made up of 3 functional domains including DNA binding domain (DBD), automodification domain (AMD) and catalytic domains (CD). The DNA binding domain contains zinc fingers that can bind to breaks in DNA and contains the nuclear localization signal (NLS), which ensures the translocation of PARP1 into the nucleus and also forms a site of cleavage by caspase 3. The AMD is responsible for addition of ADP-ribose polymers to PARP1 itself. The catalytic domain is responsible for the PARP activity [60] (Figure 1).

The DNA binding domain extends from the initiator methionine (M) to threonine (T) 373 in human PARP1, and contains 2 well known structurally and functionally unique zinc fingers (FI: amino acid 11-89; FII: amino acids 115-199) [61,62]. A recently discovered third and thus far an unrecognized zinc-binding motif, (FIII: amino acid 233-373) has been reported [63,64]. DNA binding domain contains a bipartite nuclear localization signal (NLS) of the lysine (K) rich form KRK-X-KKKSKK (amino acid 207-226) that targets PARP1 to the nucleus [65]. Zinc fingers FI and FII are thought to recognize altered structures in DNA rather than particular sequences. These zinc fingers have been reported to be involved in protein - protein interactions [66]. PARP1 strongly associates with single and double strand DNA breaks generated either directly by DNA damage or indirectly by the enzymatic excision of damaged bases during DNA repair processes.

Several studies suggest that the first zinc finger is required for PARP1 activation by both DNA single and double strand breaks, whereas the second zinc finger may exclusively act as a DNA single strand break sensor [61,62,67]. Additional studies are necessary for further identification of interactions/localization of PARP or different mutation/polymorphisms of PARP in the pathophysiology of oxidative stress, apoptosis and malignancies [68].

**Figure 2:**
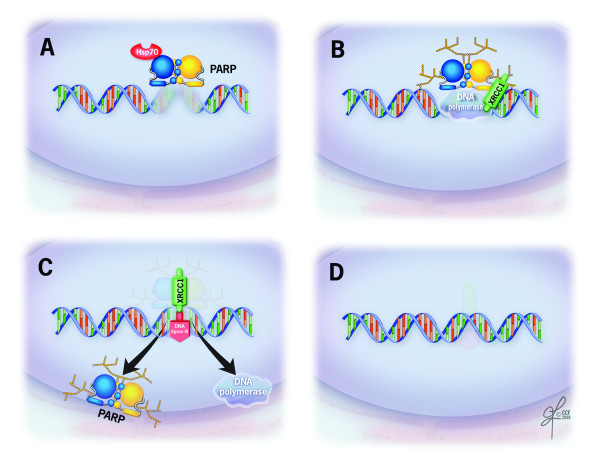
**PARP interactions in DNA damage/repair showing**. A: DNA damage caused by genotoxic agents activates PARP with HSP70 (heat shock protein 70) providing further activation B: activation of PAR formation with further help from other DNA repair proteins such as XCCR1 (X-ray repair complementing defective repair in Chinese hamster ovary cells) that help DNA polymerase to start sealing the damaged DNA strand C: DNA polymerase and ligase seal the DNA nicks releasing PARP and other DNA binding proteins and D: repaired DNA.

The major PAR acceptor protein is PARP1 itself, which appears to accumulate roughly 90% of cellular PAR via PARylation of its auto-modification domain. NAD is the substrate of PARP enzymes that becomes cleaved forming ADP-ribose and nicotinamide (Figure 2). Binding of monomer of ADP-ribose with PARP1 is mainly through hydrogen bonds on the c-terminus mainly the automodification domain [53]. Interestingly, Altmeyer et al (2009) showed that glutamic acid residue in the automodification domain of PARP1 is not required for PAR formation. Instead they identified lysine residues to be the PAR acceptor sites in PARP1 [67].

### Role of PARP in metabolic pathways

The real challenge and the difficulty in understanding PARP interaction and PAR metabolism is the lack of structural information that can be provided by X-ray crystallography or by nuclear magnetic resonance (NMR). Prior to the recent findings by Altmeyer et al [67], the intense research on PARP was unable to confirm the presence of glutamic acid residue in the AMD that may be functioning as the PAR acceptor of amino acid in PARP1 [69]. This was mainly due to the lack of mutational studies for PARP. Figure 2 explains the possible PARP interaction with the main proteins involved in DNA repair during DNA damage/repair process. The primary catalytic function of PARP enzymes is to transfer ADP-ribose groups to the glutamate, aspartate and carboxy-terminal lysine residues of proteins. With NAD serving as a cofactor, PAR polymerization begins by breaking the glycosidic bond between ADP-ribose and nicontinamide. PARP can elongate the amino acid chains of recipient proteins in a linear or branched manner by the addition of up to 200 ADP-ribose groups [70].

PARP attaches ADP-ribose groups to a variety of protein substrates. Perhaps the most common target of (ADP-ribosyl)ation is PARP1 itself, termed auto-modification [71]. PARP enzymes commonly modify nucleosome proteins in order to restructure chromatin. While both histone H1 and H2B are acceptors of poly ADP-ribose, histone H1 is the major acceptor recipient [72,73]. Histone H1t, a major H1 variant in testis, is the main H1 target of PARP during spermatogenesis [74]. PARP activity is not restricted to nuclesome proteins; the well-known tumor suppressor protein p53 is also modified by PARP1. This modification transcriptionally inactivates p53 [75]. DNA polymerases modified by PARP have also been inhibited during *in vitro *studies [76]. Toposiomerase II can also be modified by PARP activity [77]. Similarly, the transcription nuclear factor kappa β (NF-kβ) can also be modified by ADP-ribose group attachment [78].

Regulation of PARP activity is important for exploring the therapeutic options of this enzyme. Several types of molecules have been identified as activators of PARP activity including histones, a common target of PARP. Though histones H1 and H2B are modified by PARP1, histones H1 and H3 reciprocally activate PARP1 [79,80]. Apart from ribosylation, the structure of histones is regulated by acetylation and silent information regulator gene (SIRT1), a histone deacetylase, involved in the maintenance of histone structure. SIRT1 has a regulatory action on PARP1 activity and in the absence of SIRT-1, PARP remains unregulated resulting in apoptosis inducing factor (AIF) regulated cell death [81]. PARP activity is also activated by a number of metal ions (like magnesium and calcium) and polyamines. Incidentally, calcium ions also play an important role in the pathophysiology associated with oxidative stress and could provide a link to explain the effect of oxidative stress on PARP activity [82-86].

There are a number of inhibitors used to study PARP activity such as endogenous purines (hypoxanthine and inosine) or exogenous molecules like caffeine derivatives or tetracycline derivatives [80,87]. PARP1 phosphorylation by ERK1/2 is required for maximal PARP1 activation after DNA damage [88]. Furthermore DNA-dependent protein kinase (DNA-PK), a protein involved in the repair of double strand breaks results in suppression of PARP activity probably through direct binding and/or sequestration of DNA-ends which serve as an important stimulator for both DNA-PK and PARP [89].

### PARP in mammalian cells

The most important role of PARP is its capacity to repair DNA, especially in resolving single strand breaks. PARP1 and PARP2 have been shown to function in the repair of base excision [60]. Additionally, PARP1 may play a role in an alternate version of double strand DNA break repair involving the DNA repair protein XRCC1 (X-ray repair complementing defective repair in Chinese hamster ovary cells 1) along with Ligase III and DNA-dependent protein kinase involved in resealing DNA breaks [90,91]. PARP interactions and it's role in the DNA repair process is explained in Figure 2. PARP1/PARP2 get activated with DNA breaks and interact along with other main DNA repair proteins (XRCC1, DNA polymerase, DNA ligase III, and other DNA binding proteins to repair the damaged DNA strand. Heat shock protein may provide additional activation for the PAR formation early in the DNA repair [92] (Figure 2).

PARP1 binds to broken strands of DNA and automodifies itself. It then dissociates from the single DNA strand due to the negative charge it acquires from the ADP-ribose group. After dissociating, PARP associates with DNA Ligase III alpha, which is involved in the resealing of DNA breaks, and XRCC1. XRCC1 then recruits other repair factors (such as DNA polymerase β, apurinic-apyrimidinic (AP) endonuclease, and polynucleotide kinase) to complete the repair process [93]. Thus, PARP1 is part of an important signaling pathway for DNA damage repair due to its ability to recruit other repair enzymes necessary to preserve DNA integrity of a cell. The role of PARP1 in base excision repair (BER) was recognized by its interaction with DNA polymerase β and its interaction with DNA Ligase III and XRCC1. It was also shown that PARP1 deficient cells demonstrated significantly inhibited BER activity [94].

Similarly, PARP2 has also been shown to participate in BER pathways, associating with DNA polymerase β, XRCC1, and DNA Ligase III [51]. PARP2 deficient mice showed significant delays in resealing single strand DNA breaks similar to those seen in PARP1 deficient mice. This is interesting because PARP2 activity is 10 times less than that of PARP1. PARP1 and PARP2 also appear to homodimerize and heterodimerize as a part of DNA repair [51]. PARP not only serves as part of a signaling pathway in DNA damage repair but it is also involved in the repair process. In particular, the binding of PARP1 to the broken ends of DNA may protect it from degradation by nucleases [95]. NBS1 (Nijmegen Breakage Syndrome 1) has been recently reported to be required for base excision repair (BER) [96].

A model put forth recently suggests that the involvement of PARP in DNA damage repair is regulated by a feedback mechanism [97]. PARP1 is first recruited to the DNA break site and then binds to this site through its DNA binding domain. The addition of PAR units enables the recruitment of more PARP1 moieties through the AMD of PARP1. The aggregation of PARP molecules then creates a signal that recruits other repair factors and forms the positive feedback mechanism of PARP. Interestingly, PARP activity is subject to a negative feedback mechanism that prevents excessive accumulation of poly (ADP-ribose) and cell death [98,99].

### PARP in cell cycle and cell death

**Figure 3:**
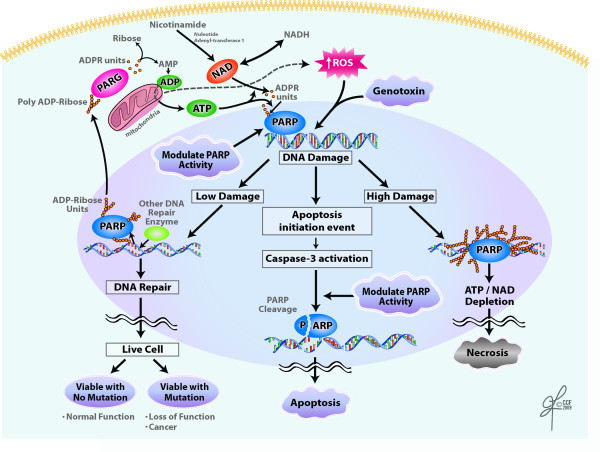
**Possible role of PARP in cell death in the event of DNA damage caused by ROS or a genotoxin, PARP targets the damaged site**. If high damage occurs, PARP may become overactivated resulting in ATP/NAD depletion and necrosis. Apoptosis can also occur through caspase-3 activation and PARP cleavage. If low damage occurs, PARP can recruit other repair enzymes and DNA repair can occur. Recently PARP1 dependent cell death termed as parthanatos has been reported, and is distinct from apoptosis, necrosis or autophagy. Each arrow represents one or more reaction(s).

In addition to being involved in the rescuing function of DNA repair, PARP1 is also directly involved [100] in both programmed cell death as well as necrosis [101-103] (Figure 3). PARP is part of the caspase-dependent pathway of apoptosis and as part of this caspase mediating pathway; PARP1 is cleaved by Caspase-3 into a 25 kDa N-terminal and an 85 kDa C-terminal fragment. The 25 kDa fragment consists of the DBD and the 85 kDa fragment consists of the AMD and CD (Figure 1). The detachment of the DNA-binding domain from the automodification and catalytic domains inactivates PARP1 and allows apoptosis to occur [104]. Meanwhile, the N-terminal fragment inhibits any uncleaved PARP1 molecules that are still present. Thus, PARP1 cleavage effectively inhibits PARP activity that could result in energy depletion as a result of consumption of a significantly large number of Nicotinamide adenine dinucleotide (NAD) molecules and lack of ribosylation [105]. PARP1 is also involved in a caspase-independent cell death pathway. When PARP1 is activated by exposing fibroblast cultures to hydrogen peroxide-induced damage, it triggers caspase-independent pathway. PARP activation triggers the release of AIF from the mitochondria and causes it to relocate to the nucleus resulting in chromatinolyis [55,106-108].

PARP is also involved in a less organized version of cell death-necrosis. Originally Berger et al proposed that depletion of NAD caused an over-activation of PARP and in the milieu of excessive DNA damage can cause necrosis [109]. Heeres and Hergenrother proposed that over-accumulation of poly (ADP-ribose) are cytotoxic to cells and induce them to undergo necrosis.

Thus PARP appears to be a two-sided coin. It has the potential to respond to threats to DNA integrity, but its over-activation can lead to cell death [101]. Figure 3 represent a schematic pathways that link the reactive oxygen species (ROS) and genotoxins induced DNA damage with the ATP/NAD levels that may determine the cell death pathway depending upon the extent of the DNA damage, caspase activation, and ATP/NAD levels. Modification of PARP activity through inhibition or cleavage may lead to apoptosis by preventing DNA repair provided by PARP. However, recent report indicate that PARP may lead to PARP1 dependent cell death that is reported to be distinct from apoptosis, necrosis or autophagy and it is called parthanatos [110].

The role of PARP in maintaining genomic integrity could have profound implications for cell division. PARP is associated with specific structures important for cell division such as centromeres and centrosomes, pointing to its possible role in cell division. PARP1 associates with centrosomes during cell division and interphase, which may be related to PARP1's role in maintaining chromosome stability [111]. PARP1 is also involved in spindle structures necessary for cell division. Poly (ADP-ribosyl)ation of spindle structures is essential for normal functioning and assembly of spindles that if disturbed could result in defective chromosome segregation [112,113]. PARP3 also demonstrated an interaction with PARP1 at centrosomes [57]. PARPs localize to active mammalian centromeres primarily during metaphase and prometaphase [114,115]. PARP1 and PARP2 bind to the same centromere proteins, including the important mitotic checkpoint protein and Bub3 (budding uninhibited by benzimidazoles 3) [55].

### Chromatin remodeling and transcription

Poly (ADP-ribosyl)ation can be considered an epigenetic modification [116] because its modification of histones can remodel chromatin structure in order to provide unique information for other proteins involved in not only DNA repair but also in transcription [55,97]. The role of PARP in transcription is a result of two important aspects of its function: its modification of histones and interaction with other coactivators and DNA binding factors to bind to the enhancer promoter regions [117-119].

The chromatin environment is very important for transcription and the role of PARPs in chromatin remodeling has been well documented [55,108]. PARP has been found to associate with the Facilitates of Chromatin Transcription (FACT) complex, a protein complex involved in chromatin remodeling. Poly (ADP-ribosyl)ation of the FACT complex prevents the interaction between FACT and nucleosomes *in vitro *[120]. As mentioned before, Histone H1 and H2B are major acceptors of PARP modification and this modification can also remodel chromatin structure [116]. Specifically poly (ADP-ribosyl)ation of nucleosome structures can cause relaxation of chromatin structure [116,121,122].

In a recent study, PARP1 was shown to be broadly distributed across the human genome. Furthermore, PARP1 and histone H1 were shown to have reciprocal roles in regulating gene expression. When there is increased presence of PARP and decreased presence of H1 at promoter regions of genes, the genes are activated in 90% of the cases. When gene promoters had both decreased presence of PARP and H1, less than 45% of genes were expressed [123]. Tulin and Spalding reported that PARP can activate the transcription of heat shock proteins in Drosophila by decondensing chromatin structure [108]. This was in contradiction to the study of Oei et al who showed that poly (ADP-ribosyl)ation of transcription factors for TATA-binding protein (TBP) or YinYang1 (YY1) prevented these transcription factors from binding DNA [124]. However, once TBP or YY1 were bound to DNA, they were immune to the action of PARP. Therefore, PARP cannot dislodge TBP or YY1 once they are bound to DNA. In this way, PARP can prevent transcription in specific parts of the genome without disturbing ongoing transcription [60,125,126].

PARP interacts with transcription factors at enhancer and promoter regions via its involvement with NF-κβ, [23,78,127,128]. PARP-NF-κβ interactions have important consequences for inflammation. PARP1 deficient mice cloned by Oliver et al were resistant to endotoxic shock normally induced by exposure to lipopolysaccaride (LPS) [102]. This could be a result of decreased expression of genes controlled by NF-κβ [35,97,126,129,130].

The epigenetic role of PARP extends to other genomic structures besides histones. Poly (ADP-ribosyl)ation of the CCCTC-binding factor (zinc finger protein) CTCF gene, a chromatin insulator encoding protein has been shown to affect the ability of this protein to interact with over 140 target sites in mice [107,131-133]. The role of PARP in controlling the function of the CTCF insulator in the regulation of the transcriptional states of various genes further demonstrates the role of PARP in epigenetics [134,135].

### Poly (ADP-ribose) glycohydrolase (PARG) and PARP interactions

Poly (ADP-ribose) glycohydrolase or PARG is an enzyme involved in poly (ADP-ribose) metabolism. PARG removes PAR units from proteins and thus plays an equally important role as PARP in cellular function. Though PARG is found in the cytoplasm rather than the nucleus where PARP is found [136], PARG can be transported between the nucleus and cytoplasm in order to regulate the breakdown of poly (ADP-ribose) [137]. It was found that a PARG deficiency in mice was lethal because of an accumulation of poly (ADP-ribose) [138].

Specifically, PARG maintains chromatin structure by removal of poly(ADP-ribose) and by acting in opposition to PARP return chromatin to its original state. PARG accomplishes this by removing PAR groups from histones and once again allowing histones to form the nucleosome structure of chromatin [137]. PARG is involved in DNA repair by regulating the amount of PAR synthesized in response to DNA damage since excessive accumulation of PAR may result in cell death [101]. PARG and PARP work in opposition to each other to modify chromatin structure [117,122]. When PARP creates transcriptionally active regions of chromatin, PARG restores chromatin to its original state. However, PARP does not always transcriptionally activate chromatin regions. For instance, in euchromatin regions, PARP is involved in chromatin decondensation and promoting transcription while in heterochromatin regions it could repress transcription [117]. Knocking out of PARG was found to be lethal in mouse embryonic cells at day 3 of gestation since PARG is the primary enzyme involved in breaking down PAR in cells [138].

Due to its abilities to regulate the ADP-ribose that is required for protection against DNA damage, PARG is involved in cellular responses to oxidative stress [139]. In a recent study by Fisher et al, PARG was found to cooperate with PARP1 in responding to oxidative damage by regulating XRCC1 at DNA break sites created by oxidative damage [140]. PARG has also been shown to protect against cell damage caused by genotoxic or oxidative stress even at sub-lethal levels by regulating PAR [138].

### Role of PARP in germ cell death

Apoptosis is a normal component of mammalian spermatogenesis. It is orchestrated spontaneously during the entire stages of spermatogenesis in order to produce mature spermatozoa and to eliminate any abnormal spermatozoa. In fact, a very large number of spermatozoa die and are eliminated during spermatogenesis. This may be due to the ability of the Sertoli cells to maintain only a limited number of germ cells and resulting in the elimination of excess germ cells. Apoptosis may also function to destroy cells that do not make it past certain cellular checkpoints [141] Evidence seems to point to the notion that germ cell death during mitotic and meiotic cell divisions may be needed to eliminate problems such as errors in chromosomal arrangement during meiosis or unrepaired breaks in DNA. More importantly, apoptosis may be needed to prevent genetic abnormalities from being passed onto offspring [142]. In a recent study, Codelia et al examined which cell death pathway was involved in pubertal rat spermatogenesis. Using a caspase-8 inhibitor and a pan-caspase inhibitor they detected significantly less cleaved PARP and also a reduction in the number of apoptotic germ cells suggesting that germ cell apoptosis occurs via the Fas antigen (Fas)-Fas ligand (Fas-FasL) system and that PARP cleavage may play a key role [143].

Not only does PARP have a well-defined role in DNA repair, but it is also involved in apoptosis. During apoptosis, numerous DNA strand breaks can lead to PARP activation. This activation of PARP may be an attempt by the dying cell to repair the DNA damage caused by nuclease activation [129,144,145]. However, this attempt to repair damage proves futile as PARP is cleaved by caspase-3 into a catalytic fragment of 89 kDa and DNA binding unit of 24 kDa [61,146]. Therefore, this cleaved version of PARP could be a biochemical marker of caspase-dependent apoptosis [147,148].

Deregulation of germ cell death can have important implications for male fertility. Patients with contralateral testes exhibit an increased incidence of apoptosis. The presence of apoptotic markers is high in these patients especially in spermatocytes, early and late spermatids, and Sertoli cells [149]. Infertile men with spermatid and spermatocyte maturation arrest and hypospermatogenesis also show increased apoptosis [150]. Specifically, the Fas-FasL pathway and active caspase 3 showed increased activity in testes with maturation arrest and Sertoli cell-only syndrome (SCOS) [127,151]. Increased rate of apoptosis seen with these infertility cases may be due to elimination of germ cells with extensive DNA damage. Tesarik et al compared men with complete spermiogenesis failure to another group of azoospermic men who had incomplete failure of spermiogenesis and were able to show that apoptotic DNA damage was greater in the latter group when compared to the former. This increase in DNA damage seen in patients with complete spermiogenesis failure could be responsible for the low conception success rates in these cases [152]. In a more recent study, Maymon et al proposed that the presence of greater PAR levels in human spermatocytes during maturation arrest could be correlated with the greater occurrence of DNA strand breaks during impaired spermatogenesis [153].

PARP2 has also been implicated in abnormal spermatogenesis. In a recent study by Dantzer et al, PARP2 deficient male mice were found to have hypofertility [154]. Upon examination of infertile PARP2 null mice, an increased incidence of testicular apoptosis was found specifically in the spermatocyte and spermatid layers. However, the layers containing spermatogonia and preleptotene spermatocytes were normal. Chromosome segregation was abnormal during metaphase I and spindle assembly was also abnormal in these PARP2 deficient mice. Thus, the decrease in fertility seen in these PARP2-null mice could be related to both defective meiosis I and spermiogenesis [81]. These results make it increasingly clear that apoptotic markers can be excellent diagnostic tools for evaluating fertility potential.

Exogenous agents applied to the testes may also activate caspase-dependent cell death pathway. For example, when the scrotal temperature was increased in rats over time, the mitochondria dependent cell death pathway in the testis was activated. The signaling cascade involved relocation of Bax, translocation of cytochrome C, activation of caspases, and the cleavage of PARP [155]. Although the precise role of PARP in exogenously induced apoptosis is not clear, a study of PARP's protein targets may help elucidate this role. Following exogenous stress, increase in levels of p53 are considered to be a part of the mechanism that returns spermatogenesis to normal cycles following apoptosis. P53 is a downstream protein poly (ADP-ribosyl)ated by PARP [71,75,156]. Under conditions of inflammation, PARP poly (ADP-ribosyl)ates and activates NF-κβ in human Sertoli cells [157]. Treatment with anti-inflammatory agents suppress this cell death pathway thereby validating the involvement of PARP in inflammatory responses [158,159]. It remains to be seen if this anti-inflammatory activity of PARP could also be applied to germ cells.

### PARP and spermatogenesis

PARP1 has been detected in the nuclei of a variety of tissue types including the brain, heart, kidney, and testis [121]. PARP2 has been detected in the liver, kidney, spleen, adrenal gland, stomach, intestinal epithelium, thymus, brain tissue, and testis. PARP2 expression is weaker than that of PARP1 and is well distinguished [51]. PARP1 has high levels of expression in the basal regions of seminiferous tubules of developing mice, but has almost no presence in the luminal region of the seminiferous tubules, suggesting that PARP1 is down-regulated during the haploid stage of meiosis [51]. This explains why PARP is expressed significantly during the earlier stages of spermatogenesis. However other reports show that in the rat, the highest concentrations of PARP1 are seen in primary spermatocytes followed by spermatids as transcription declines in late stages on maturation [160,161]. In a study by Tramontano et al examining rat primary spermatocytes it was found that both PARP1 and PARP2 are present in these germ cells. However, the vast majority of PAR in these rat primary spermatocytes was produced by PARP1 suggesting possibly different roles of PARP1 and PARP2 in spermatogenesis [107]. Interestingly, PARG has also been detected in the nuclei of rat primary spermatocytes [160] suggesting the presence of a mechanism to regulate the levels of poly (ADP-ribose) in germ cells.

In a recent study using human testicular samples, it was shown that that the strongest levels of PARP1 were found in spermatogonia. Presence of poly (ADP-ribose) differed slightly with the stage of spermatogenesis. Poly (ADP-ribosyl)ation was strongest in human round and elongating spermatids as well as in a subpopulation of primary spermatocytes. In contrast, mature spermatids had no PARP expression or poly (ADP-ribosyl)ation [153]. This is in accordance with a study in rat germ cells where poly (ADP-ribose) and NAD levels progressively decreased from primary to secondary spermatocytes and to a greater extent in spermatids [74]. This decrease in PARP1 levels and activity throughout differentiating male germ cells may be correlated with the changes in chromatin structure associated with spermatogenesis. The chromatin remodeling steps of spermatogenesis include the replacement of histones by protamines [162] and a transition from a supercoiled form of DNA to a non-supercoiled form [163]. It is during these chromatin remodeling steps of spermiogenesis that DNA strand breaks can occur. In human testis, an increase in DNA strand breaks occurs in 100% of elongating spermatids [164]. These breaks were later demonstrated to be double stranded breaks caused by topoisomerase II as a result of the unique chromatin packaging steps that take place during spermatogenesis [14].

Meyer-Ficca et al (2005) demonstrated the presence of poly (ADP-ribose) (PAR) in elongated spermatids of rat [39]. They showed that during these steps when a high number of DNA breaks occur directly preceding nuclear condensation, there is correspondingly a higher amount of PAR in rat germ cells. Greater PAR formation through PARP1 and PARP2 action occurs during this phase of spermatogenesis that includes a great deal of chromatin condensation (steps 11-14 of rat spermatogenesis), PAR levels decrease only when protamines appear in the chromatin. Thus, PAR formation could be important for repairing DNA strand breaks during these crucial chromatin remodeling steps of spermatogenesis [39,165]. Furthermore, PAR formation could also be important for histone modification because not only is there auto-modification of PARP during spermatogenesis, but much of PARP activity is targeted towards the testes-specific histone, H1t.

The activation of Histone 2A (H2AX), a biological marker of DNA breaks, and the poly (ADP-ribose)ylation of histones at break sites may act as markers of such damage [166]. Thus, the activity of PARP during the chromatin remodeling steps of spermatogenesis in terms of repairing double stranded breaks and the poly (ADP-ribosyl)ation of histones, is critical and disregulation of the chromatin remodeling steps of spermiogenesis could have serious consequences for the male gamete [164]. PARP2 knockout mouse was shown to be associated with severely compromised spermatids and delays in elongation process [154]. Heat stress has been reported to decrease PARP expression in the rat testis [50]. However heat shock protein was reported to activate the PARP and PAR formation [92].

The quest to detect PARP in ejaculated spermatozoa has met with success only recently. Taylor et al did not detect the presence of PARP1 in human ejaculated sperm samples when analyzing semen for apoptotic markers [167]. However, in a recent study by Jha et al, [168] several isoforms of PARP were detected in ejaculated spermatozoa including PARP1, PARP2, and PARP9. Immunolocalization patterns showed that PARP was found near the acrosomal regions in sperm heads. Furthermore, a direct correlation was seen between sperm maturity and the presence of PARP, i.e., an increased presence of PARP1, PARP2, and PARP9 was seen in mature sperm when compared to immature sperm. Higher levels of PARP1, PARP2 and PARP9 were seen in ejaculated sperm from fertile men when compared to infertile men indicating a possible relationship between PARP and male infertility. PARP activity was modulated to determine its role in the response to oxidative and chemical damage in sperm. In the presence of a PARP inhibitor, 3-aminobenzamide, chemical and oxidative stress-induced apoptosis was reported to increase by nearly two-fold. This novel finding suggests that PARP could play an important role in protecting spermatozoa subjected to oxidative and chemical damage [168].

### PARP and sperm DNA

Recently the importance of DNA damage to sperm of infertile men has gained attention. It has been shown that that a higher index of DNA damage can possibly lead to lower semen quality. In a study involving 322 couples, when DNA fragmentation exceeded an index of 15%, there was increased incidence of non-transfer and miscarriages after performing ICSI [169]. Studies such as these warrant the investigation of factors that may be responsible for maintaining genomic integrity especially in ejaculated spermatozoa. DNA damage repair through PARP activity has been demonstrated in rat germ cells. Atorino et al showed that after extensive DNA damage in rat spermatids and spermatocytes caused by radiation and ROS damage *in vivo*, a PARP inhibitor caused a delay in DNA damage repair [40]. They were able to demonstrate up to a 3-fold increase in PARP activation as cells recovered from these damaging agents [60,125,126]. The main difference between spermatids and primary spermatocytes was that only spermatids showed detectable PAR production after genotoxic stress. Atorino et al hypothesized that primary spermatocytes did not show the same degree of response possibly due to the different chromatin states of these two germ cells. Thus, PARP activity in response to genotoxic stress may be important for preventing mutations from accumulating and being passed on to offspring [40].

Though the role of PARP in repairing DNA damage in ejaculated spermatozoa has yet to be thoroughly investigated, it has been found that DNA damage caused by sperm cryopreservation can be repaired through PARP activity. In a study by Kopeika et al, cryopreserved sperm from loach (fresh water fish related to carp) were used to fertilize eggs and the embryos were exposed to a PARP inhibitor [170]. It was found that survival was significantly decreased in embryos exposed to PARP inhibitors when compared to control. This study suggests a possible role for PARP in repairing paternal DNA damage and it also showed that it was possible for the oocyte to repair this damage even in presence of PARP inhibition [170].

Cryopreservation is not the only threat to genomic integrity. It is still controversial whether malignancy itself is the cause of chromatin damage in mature spermatozoa. In a study analyzing 75 men with various types of testicular and non-testicular cancers, the degree of DNA fragmentation did not differ between types of cancer. But surprisingly, the levels of DNA fragmentation in cancer patients were similar to the levels found in infertile men [171]. Another study found that patients with testicular cancer and Hodgkin's Lymphoma were normospermic, but had increased levels of DNA damage along with decreased chromatin compaction [172]. However, a third study in the same year hypothesized that malignancy alone was not responsible for increased DNA damage seen in sperm. This study showed that thawed sperm samples from cancer patients had similar levels of DNA fragmentation to that of sperm with low freezability collected from healthy donors. This study attributed the increased sperm DNA damage seen in the decreased freezability of these semen specimens and not to malignancy [173].

Nonetheless, cancer patients are advised to freeze sperm prior to therapy as an option to preserve their fertility potential. Patients undergoing radiotherapy have shown a temporary increase in the sperm with DNA strand breaks and a decrease in fertility despite having normal sperm concentrations [42]. Chemotherapy does not produce the same deleterious effects as radiotherapy in its initial phase. Decrease in sperm DNA fragmentation is generally seen after 3 or more cycles of chemotherapy [174]. Chemotherapy (especially alkylating agents) and radiotherapy, even in low doses, can damage the seminiferous epithelium and impair spermatogenesis in both children and adults [175].

Apart from treatment by either chemo- or radiotherapy, malignancy itself causes a significant threat to sperm DNA damage. This could mean that cryopreservation of sperm prior to therapy may not be sufficient to preserve fertility [174]. Instead, therapeutic options such as modification of PARP activity could be used in order to retain genomic integrity under threats of malignancy and radiotherapy to eliminate the low quality spermatozoa with DNA damage [176,177].

### Sperm DNA damage repair defects

DNA polymerase activity in non-replicating cells is associated with DNA repair. Consequently, increase in apoptotic markers seen in the semen of infertile men may also be an indicator of increase DNA repair activity [15]. Evidence from the literature indicates that DNA repair systems may play a role during spermiogenesis. Elements of base excision repair (BER) have been identified in elongated spermatids [178]. Mismatch repair (MMR) involving the mutS homolog 2 (MSH2) proteins has a role in spermiogenesis. Interestingly, in a mouse model for Huntington disease, deletion of MSH2 in Huntington disease abolished trinucleotide cytosine-adenine-guanisine (CAG) repeat expansion between round spermatids and spermatozoa. CAG, is a DNA mutation responsible for causing any type of disorder categorized as a trinucleotide repeat disorder. Presence of CAG repeat expansion may explain the earlier onset of the disease and the severity of the symptoms through successive generations of an affected family due to the expansion of these repeats. Deletion of MSH2 suggests an active role for MSH2 during extensive DNA repair [179-182]. Quite interestingly, caffeine may lead to inactivation of H2AX and non-homologous end-joining (NHEJ) DNA repair [183]. Impairment in DNA repair during spermiogenesis may result in persistent double stranded breaks in mature spermatozoa. Further investigation may provide important clues regarding the consequences of the endogenous DNA strand breaks and repair in spermatids and mature spermatozoa [184].

Down regulation of DNA repair genes such as Ogg1 (involved in base excision repair), Rad54 (involved in double-strand break repair) and Xpg (involved in nucleotide excision repair) has been reported using global genome expression by DNA microarray following exposure to heat stress at 43°C [30]. Heat stress induced by cryptorchidism appears to result in decreased expression of DNA polymerase B and DNA ligase III both of which are involved in the final stages of DNA repair [185,186].

### PARP - a new marker in ejaculated spermatozoa

The role of PARP in DNA repair (Figure 2) and its presence during stages of spermatogenesis suggest that it is involved in maintaining genomic integrity in ejaculated sperm. However, the presence of PARP has only recently been shown in ejaculated sperm samples. Furthermore, Jha et al have suggested a correlation with the presence of PARP and male fertility [168]. This study suggested that the decreased presence of PARP in the sperm of infertile men could be the cause of increased DNA damage seen in poor quality semen samples. DNA damage repair in germ cells as mediated by PARP is therefore as yet unexplored confounder of male fertility. Further studies are needed to fully explore the role of PARP in DNA repair especially in reproductive medicine [50-52,55,187]. The existing methods of detecting infertility from semen samples are quantitative methods involving the count and observation of sperm and are dubious due to the constantly changing 'normal' seminal values. A qualitative method would thus serve as a more reliable method of detecting infertility [125,162]. In view of this, the detection of apoptotic markers such as caspase and phosphatidylserine (PS) have been successfully correlated with a variety of infertility conditions; however, the use of PARP as an apoptotic marker has not been fully investigated.

Taylor et al found greater caspase activity in low motility sperm samples from infertile men when compared to those with high motility [167]. The active caspase enzymes have been localized in the human spermatozoa predominantly in the post acrosomal region (caspase 8, 1 and 3) [188-190] or in the mid-piece [191]. A significant positive correlation between in-situ active caspase-3 in the sperm midpiece and DNA fragmentation was observed in the low motility fractions of patients, suggesting that caspase-dependent apoptotic mechanisms could originate in the cytoplasmic droplet or within mitochondria and function in the nucleus [192]. These data suggest that in some ejaculated sperm populations, caspases are present and may function to increase PS translocation and DNA fragmentation. Furthermore fluorescence staining of active caspases localized the enzymes mainly to the postacrosomal region in sperm from donors. This pattern differed slightly from that of patients, in whom additional cytoplasmic residues were found to be highly positive [193].

In an interesting study published by Falerio and Lazebnik in 2000, question as to how caspase 3 which is usually cytoplasmic gains access to its nuclear targets was examined [194]. These investigators suggested that caspase-3 was actively transported to the nucleus through the nuclear pores. They found that caspase-9, which is activated earlier than caspase-3, directly or indirectly inactivated nuclear transport and increased the diffusion limit of the nuclear pores. This increase allowed caspase-3 and other molecules that could not pass through the nuclear pores in living cells to enter or leave the nucleus during apoptosis by diffusion. Hence they suggested that caspase-9 contributes to cell disassembly by disrupting the nuclear cytoplasmic barrier [194].

Unlike somatic cells, early studies were not able to detect PARP1 or AIF (which is activated by PARP) in ejaculated sperm [106,167,195,196]. Similarly, the presence of cleaved PARP could not be detected in ejaculated sperm [197]; although PARP activity was demonstrated in the testis [198]. We studied the localization of the PARP in mature and immature spermatozoa in fertile and infertile men [168]. Mahfouz et al were the first to demonstrate the presence of cleaved PARP in ejaculated spermatozoa. When these sperm samples were exposed to PARP inhibitors after chemical and oxidative stress, there was a decreased incidence of apoptosis [199]. Although these authors did not study PARP and caspase 3 co-localization, they proposed that PARP cleavage may occur by activated caspase 3 located in the post acrosomal region. It would be worthwhile to replicate these findings in other mammals and investigate the use of cleaved PARP as a diagnostic tool to predict/detect male infertility.

### PARP and oxidative stress

Oxidative stress (OS) occurs when there is an increase in ROS levels and/or a decrease in the activity of the antioxidant enzymes that scavenge these harmful free radicals [200,201]. Such conditions of oxidative stress arise when germ cells are faced with biological (such as lipopolysaccharide, LPS) or chemical stressors (e.g. environmental toxicants, endocrine disruptors etc.) [20,31,202-204]. The extent of oxidative stress induced depends on the dose and duration of exposure to the stressor [204]. Oxidative stress can cause modification of proteins associated with developing spermatozoa and cause the premature release of sperm from seminiferous tubules [205]. Even a transient state of oxidative stress, spanning a few hours has been shown to bring about protein changes and stimulate a caspase-3-mediated cell death pathway and apoptosis [43,206]. In the reproductive milieu, oxidative stress is also linked to inflammation particularly since inflammatory cytokines dramatically arrest spermatogenesis and may lead to infertility [207]. However, the most important effect is the ability of oxidative stress to cause DNA damage. PARP responds to all three of these changes that can occur in the cell as a result of oxidative damage. However, there is a great deal of variability in PARP activation as a result of this type of stress depending on the metabolic stage of the cell or its microenvironment [208-210]. As a result of its interaction with NF-κβ, PARP has an important role in the inflammation process [78,102]. In response to DNA damage caused by oxidative species, PARP1 recruits the DNA repair protein XRCC1 to the sites of the damage [211]. In addition to causing damage to DNA, oxidative species can harm histones wherein PARP activates the 20S proteosome involved in breaking down oxidatively damaged histones. Also, in response to oxidative stress caused by exposure of histones to hydrogen peroxide, a complex is formed by the binding of PARP, poly (ADP-ribose), and the nuclear proteosome [133,212]. This complex formation could be important because a condensed chromatin structure may protect DNA from strand breaks induced by hydroxyl radicals [196,213].

In conditions of oxidative stress-induced necrosis in Bax-/- Bak-/- (a proapoptotic protein that regulates the intrinsic apoptotic pathway) mouse embryonic fibroblasts, there is an activation of PARP1. PARP1-catalysed poly (ADP-ribosyl)ation causes a depletion of ATP, which promotes the autophagy of these necrotic embryonic cells [214]. PARP1 has been implicated in the repair of DNA damaged by estradiol in human estrogen-receptor-negative (ER-/-) breast cancer cells. Treatment of these breast cancer cells with 2,3,7,8-tetrachlorodibenzo-*p*-dioxin (TCDD), which is itself an estrogenic toxicant, altered the expression of enzymes responsible for the bio-activation of estrogen leading to DNA damage, PARP1 activation and DNA repair. Thus, the apoptosis of human ER -/breast cancer cells was prevented by TCDD-induced activation of PARP1 and aided the survival of these cancer cells [215]. Interestingly, administration of TCDD caused the expression of cDNA encoding a 75 kDa protein that had sequence similarity with PARP. This protein, renamed as TCDD-inducible PARP (TiPARP) possessed catalytic activity similar to PARP [216]. However, the role of TiPARP on chromosome stability, DNA repair and apoptosis are yet to be elucidated.

### PARP and ageing

The ageing process also takes its toll on DNA, which can in turn, affect the fertility potential of a male. As discussed earlier, PARP is also involved in ageing through its role in immune responses, telomere maintenance, DNA repair, spindle assembly, and cell death [217]. In a recent study, El-Domyati et al collected sperm samples from fertile men of various age groups and quantified the differences in PARP1 presence and PARP activity [198]. The expression of PARP1 and its DNA repair partner, XRCC1 were higher in spermatocytes of older men while the Sertoli cells of these men showed higher levels of PAR. Apoptosis was increased in older men who showed more active caspase-3 and cleaved PARP1 in the spermatogonia and spermatocytes. This general increase in PARP1 and DNA repair enzymes could be associated with the declining DNA integrity as a result of age [198].

Chromosomal stability is vital for the survival of an organism and chromosomal instability increases with the age of an organism and is considered a symptom of ageing. PARP is essential for chromosomal stability through its role in DNA repair. PARP activity may influence ageing by maintaining genomic stability through DNA repair, telomere maintenance, spindle stability, and cell death [209,217-219]. Under physiological conditions, both PARP1 and PARP2 affect telomere functioning by binding to telomere repeat binding factor II (TRFII) and affecting its ability to bind to telomere regions [220-222]. PARP1 is also found at telomere regions of DNA that is damaged by genotoxic agents and it may play a role in preventing damage to genomic stability [221]. PARP1 deficient mice showed greater incidence of chromosomal aberrations, polyploidy and telomere shortening. When PARP was reintroduced in the form of cDNA, chromosomal integrity was restored [223,224].

### Biological role of PARP in male fertility

PARP plays a crucial role in maintaining genomic integrity in a variety of cell types and perhaps nowhere is this genomic integrity more important than in germ cells. Cases of male infertility are associated with abnormal sperm chromatin and DNA structure. The problems that arise in genomic integrity of sperm come from a variety of sources including spermatogenesis defects, abortive apoptosis, problems with spermatid maturation, and oxidative stress [22,225]. Problems in spermatogenesis could include double strand breaks that are not resolved after crossing over during meiosis I [22]. Although there has not been any clear association between apoptotic markers and DNA fragmentation in mature male gametes, it is possible that incomplete apoptosis could be a cause of such DNA fragmentation [22,226]. Maturation of spermatids involves chromatin remodeling steps that involve necessary DNA strand breaks and thus could be a source of unresolved breaks. Lastly, ROS levels and oxidative stress has been extensively investigated in male infertility, and in light of the activation of PARP-induced apoptosis pathways in oxidative stress conditions, may provide an explanation for the role of PARP in male fertility.

Thus, at the time of writing this review, the role of PARP in male fertility is not as well defined as its role in other cellular processes. However, there is enough evidence such as detection of PARP in the testis, during spermatogenesis, and in ejaculated spermatozoa to suggest that such a role exists [168,199]. Furthermore, the role of PARP as an important DNA repair enzyme could empower it with maintaining the genomic integrity of sperm. Similarly, the role of PARP in cell death pathways may have important implications for its role in the elimination of abnormal spermatozoa during the processes of spermatogenesis [227].

### Potential therapeutic applications of PARP

The key role of PARP in cell death has made it an attractive candidate in cancer therapies [157]. It is based on the simple idea that inhibiting DNA repair in malignant cells exposed to chemotherapy will kill off these cancerous cells due to the large amounts of DNA damage that will accumulate if PARP is inactivated [228]. Non-malignant cells will not be susceptible to cell death at these low doses of chemotherapy. Thus PARP inhibitors could provide a means to sensitize cancerous cells to chemotherapy and be developed as an adjuvant to chemotherapy [229]. An alternate strategy being explored is that of inactivating PARP through cleavage to attain the same end result, i.e., accumulation of damaged DNA in cancerous cells causing them to die faster [227,230].

PARP modulation may not only prove useful in cancer therapies, but also in dangerous inflammatory processes. The role of PARP in inflammation especially through the recruitment of NF-κβ and through its role in responding to oxidative stress produced by the inflammatory processes make it a powerful target for anti-inflammatory therapy [157]. The use of PARP inhibitors as therapeutics in conditions such as cerebral ischemia and other inflammation-induced conditions may be explored [159,219]. It still remains to be seen whether PARP can provide a therapy for male infertility. PARP inhibition may protect against chemically induced injury of ejaculated spermatozoa in vitro, but is not effective against damage induced by oxidative stress [199]. It is also possible that PARP inhibition may have a potential role in testicular cancer as well as cancer that may have spread to the testes [109]. Inflammatory processes as result of infections could also be another area to explore in terms of PARP and male fertility.

## Conclusion

PARP homologues have diverse role(s) in spermatogenesis and in ejaculated sperm. PARP expression is associated with sperm maturity in proven fertile men. Morphogenesis and changes during the spermatid stages result in removal of the cytoplasm from the fully mature, functionally active spermatozoa. However, many proteomic studies, other than those published by our group (Jha et al 2009) have shown differences in protein expression in spermatozoa from infertile males. Although the mature spermatozoa are transcriptionally inactive, some reports suggest that there may be enough mRNA in these mature spermatozoa, although their exact function and role is unclear and is being investigated. PARP inhibition may be used as an in vitro treatment in certain conditions such as oxidative stress and/or chemically induced death of spermatozoa with damaged DNA. Therefore these spermatozoa may not have the ability to fertilize or produce healthy embryo as the oocyte repair system may be inadequate to correct such high DNA damage. PARP modulation using kinase activators or inhibitors may have a future beneficial role in infertile patients exhibiting sperm DNA damage. This could pave the way for future studies to elucidate the role of PARP in other conditions resulting in sperm DNA damage. Cleaved PARP, which is activated during apoptosis, could serve as an apoptotic marker for differentiating healthy spermatozoa from apoptotic ones. In addition, the anti-tumor properties of PARP could provide new strategies to preserve fertility in cancer patients even after genotoxic stresses like radiation. The possibility of using DNA damaged sperm in ART especially in ICSI needs careful evaluation. PARP may hold the key to a better understanding of these repair mechanisms inherent in spermatozoa and the importance of such mechanisms in producing healthy pregnancies.

## List of abbreviations

AIF: Apoptosis inducing factor; AMD: Auto modification domain; ADPR: ADP ribose; AP: Apurinic-apyrimidinic endonuclease; ART: Assisted reproductive technologies; BER: Base excision repair; BRCA-1 CT: Breast cancer-1, human oncogene, C-Terminus; Bub3: Budding uninhibited by benzimidazoles 3; CAG: Tri-nucleotide (Cytosine- Adenine- Guanine) CD: Catalytic domain; CTCF: CCCTC-binding factor (zinc finger protein); DBD: DNA binding domain; DBD: DNA binding domain; ERK: Extracellular signal-regulated kinases; Fas-FasL, Fas antigen (Fas)/Fas ligand (FasL); H2AX: One of several genes coding for histone H2A; ICSI: Intracytoplasmic sperm injection; LPS: Lipopolysaccaride; MALDI-TOF-TOF: Matrix-assisted laser desorption/ionization time-of-flight/time-of-flight; MSH2: MutS homolog 2; NAD: Nicotinamide adenine dinucleotide; NBS1: Nijmegen Breakage Syndrome 1; NF-κβ: Nuclear factor-kappa β; NHEJ: Non-homologous end-joining; NLS: Nuclear localization signal; PAR: Poly (ADP-ribose); PARG: Poly (ADP-ribose) glycohydrolase; PARP: Poly (ADP-ribose) polymerase; Rad54: A gene linked to chromosome 1p32, encodes for a protein known to be involved in the homologous recombination and repair of DNA; ROS: Reactive oxygen species; SCOS: Sertoli cell only syndrome; SIRT1: Silent information regulator gene in human affect the metabolism and inflammation; TBP: TATA-binding protein; TCDD: 2, 3, 7, 8-tetrachlorodibenzo-p-dioxin; TRFI-I: Telomere repeat binding factor II, I; WWE domains, Domain associated with protein-protein interaction; XRCC1: X-ray repair complementing defective repair in Chinese hamster cells 1; YY1: YinYang1.

## Competing interests

No financial competing interests (political, personal, religious, ideological, academic, intellectual, commercial or any other) to declare in relation to this paper.

## Authors' contributions

AA provided substantial contribution ranging from study idea, design, information collection, critical review of the final paper. RZM participated in the original idea, medline search, drafting and finalizing the paper. RKS conceived the study, participated in the study design compilation of the contents and critical review of the paper. OS participated in compilation of the information and critical review of the paper. DM carried out the literature search, compilation of the information. PPM participated in the design of the study and helped finalize the paper. All authors read and approved the final manuscript.
